# Gene expression profiles associated with depression in patients with chronic hepatitis C (CH-C)

**DOI:** 10.1002/brb3.72

**Published:** 2012-07-06

**Authors:** Aybike Birerdinc, Arian Afendy, Maria Stepanova, Issah Younossi, Ancha Baranova, Zobair M Younossi

**Affiliations:** 1 Center for Liver Disease, Inova Health SystemFalls Church, Virginia; 2 School of Systems Biology, College of Science, George Mason UniversityFairfax, Virginia; 3 Betty and Guy Beatty Center for Integrated Research, Inova Health SystemFalls Church, Virginia

**Keywords:** Depression, hepatitis C, interferon, ribavirin, TGFβ1, Th1/Th2 cytokines, treatment

## Abstract

The standard treatment for CH-C, pegylated interferon-α and ribavirin (PEG-IFN + RBV), is associated with depression. Recent studies have proposed a new role for cytokines in the pathogenesis of depression. We aimed to assess differential gene expression related to depression in CH-C patients treated with PEG-IFN + RBV. We included 67 CH-C patients being treated with PEG-IFN+RBV. Of the entire study cohort, 22% had pre-existing depression, while another 37% developed new depression in course of the treatment. Pretreatment blood samples were collected into PAXgene™ RNA tubes, the RNAs extracted from peripheral blood mononuclear cells (PBMCs) were used for one step RT-PCR to profile 160 mRNAs. Differentially expressed genes were separated into up- and down-regulated genes according to presence or absence of depression at baseline (pre-existing depression) or following the initiation of treatment (treatment-related depression). The mRNA expression profile associated with any depression and with treatment-related depression included four and six genes, respectively. Our data demonstrate a significant down-regulation of TGF-β1 and the shift of Th1-Th2 cytokine balance in the depression associated with IFN-based treatment of HCV infection. We propose that TGF-β1 plays an important role in the imbalance of Th1/Th2 in patients with CH-C and depression. With further validation, TGF-β1 and other components of Th1/Th2 regulation pathway may provide a future marker for CH-C patients predisposed to depression.

## Introduction

Chronic hepatitis C virus (HCV) is believed to affect approximately 170 million people worldwide extending across all economic and social groups (Armstrong et al. 2006). Since a large proportion of HCV-infected individuals are currently undiagnosed, the number of newly diagnosed patients with HCV and related liver disease is expected to grow. In fact, the proportion of chronic hepatitis C patients with cirrhosis is expected to reach 25% in 2010 and 45% in 2030 (Davis et al. 2010). The considerable burden of HCV on the health care system is further compounded by the fact that HCV-related cirrhosis is the most common indication for liver transplantation (Tan and Lok 2007).

The current standard treatment for HCV is a combination of pegylated interferon-α and ribavirin, administered over a 24- or 48-week course. Despite advances in treating HCV, even with the optimal delivery of the current interferon-based regimen for HCV, only about 50% of treated patients with genotype 1/4 successfully clear the virus (Mauss et al. 2011). The success of the current treatment is multi-factorial and depends on a combination of several host, viral, and treatment factors. Viral factors consist of HCV genotype, pretreatment viral load, and presence of viral quasi-species (Timm and Roggendorf 2007). Host factors include presence of co-morbidities such as obesity, cirrhosis, ethnic background, gender, and age (Bondini and Younossi 2006; Chen et al. 2007; Neumann-Haefelin et al. 2007; Sharma et al. 2007). Finally, treatment-related factors affecting response include adequate duration of treatment, patient adherence and, importantly, optimal management of PEG-IFN and RBV-related side effects (Mulhall and Younossi 2005; Sharma et al. 2007). Consequently, there are two main areas of focus to develop future treatment regimens for HCV. One of them focuses on new therapeutics that can potentially increase the rates for sustained virological response (SVR) by developing regimens that would include direct acting antiviral agents (DAA). The other, equally important area, is to optimize treatment regiments and reduce its side-effect profile.

Despite diligent efforts by clinicians and clinical investigators, successful management of treatment-associated side effects remains a substantial problem and contributes significantly to treatment discontinuation or dose reduction (10 and 35%, respectively) (Manns et al. 2001; Dan et al. 2006). Depression disorder is one of the least tangible, and one of the most difficult IFN-related side effects to quantify in the treatment of HCV. IFN-α-induced depression is markedly similar to major depressive disorder (MDD) and may be manifested as depressed mood, irritability, emotional lability, agitation, fatigue, apathy, anhedonia, anorexia, psychomotor retardation, sleep disturbance, sexual dysfunction, memory impairment, and diminished ability to concentrate (Valentine et al. 1998). Due to the variability of the symptoms and lack of unification in their measurements, studies on IFN-α-induced depression also produce variable results with incidence rates ranging from 16 to 45% in patients receiving treatment (Fattovich et al. 1996; Hauser et al. 2002; Dieperink et al. 2003).

The most common risk factors for IFN-α-induced depression are related either to treatment regimen itself (i.e., higher dose and longer duration of medication) (Capuron and Ravaud 1999; Hauser et al. 2002; Dieperink et al. 2003; Capuron and Miller 2004) or to intrinsic factors predisposing patients to the development of DSM-IV symptom criteria for MDD. The most common risk factor of latter kind is pre-existing psychiatric problems or previously diagnosed MDD. Importantly, even subclinical depression and/or anxiety at the baseline, greatly increases the chances for the development of depression during treatment with IFN-α (Van Thiel et al. 1998; Capuron and Ravaud 1999; Fontana et al. 2002; Hauser et al. 2002; Dieperink et al. 2003; Capuron and Miller 2004). Information about “predisposition to depression” can help clinical practitioners to make some difficult decisions to more closely follow or “pre-treat” patients with a risk factor for depression with an antidepressant. This solution is imperfect, as at least 50% of patients do not suffer from any depressive symptoms during treatment, and are thereby being needlessly exposed to antidepressants. Therefore, a search for quantitatively measurable markers of IFN-α-induced depression could aid in identifying patients who would benefit from antidepressant pretreatment.

One approach is to screen for molecules connected to the pathological process of depression. IFN-α is a potent pro-inflammatory cytokine that acts to increase the serum concentrations of various other cytokines including interleukin IL-1, IL-6, tumor necrosis factor-α (TNF-α), IL-2, and IFN-α (Taylor and Grossberg 1998). Some studies have recently proposed a pivotal role for cytokine imbalance in the etiology of depression; in particular, the relevance of the Th1/Th2 cytokine imbalance in the brain during both psychological stress and with psychiatric disorders was discussed (Myint and Kim 2003). In this study, we examine the baseline expression of 153 cytokine response-related genes in patients undergoing HCV treatment and correlate our findings to treatment-induced depression symptoms.

## Methods

### Study cohort

This study cohort comprised of HCV-infected patients scheduled for treatment with PEG-IFN and RBV. Prior to treatment, clinical, demographic, and laboratory data, as well as blood samples were collected. The MDD was diagnosed according to DSM criteria at baseline and during treatment. Dose and duration of anti-HCV treatment were determined by genotype and “on-treatment” response pattern. From pretreatment blood samples, mRNA was extracted and relative gene expression levels were calculated as previously described (Garcia et al. 2005; Younossi et al. 2009).

## Statistical analysis

Differentially expressed genes were separated into up and down-regulated gene lists according to the presence of depression at baseline (Pre-existing Depression) as well as to the development of depression during treatment (New Depression). Both gene lists were subjected to intensive literature searches to determine potential associations with depression. Using both data sets, predictive models for depression were designed. Clinical parameters were compared using the chi-square test, and gene expression levels were compared using the Mann–Whitney non-parametric test. Regression models for predicting any and new depression (with these parameters used as dependent variables) were generated by stepwise bi-directional selection. Only genes that demonstrated a trend to statistical significance (*P* ≤ 0.05 before multiple test correction) were used for stepwise selection. Bi-directional selection started with a full model that contained all the genes with significantly different expression levels (based on Mann–Whitney's outputs) and clinical parameters, and ended when no more improvement (estimated using coefficient of determination) of a depression-predicting model containing TGFβ1 gene was achieved with the addition or removal of any clinical or any other gene predictor The predictive performance that included sensitivity, specificity, positive, and negative predictive values and the area under the ROC-curve (AUC) was evaluated for the generated models using ten-fold cross-validation. All analyses were run using SAS 10.2 (SAS Institute, Cary, NC).

## Results

### Demographic and clinical data

The study included 67 CH-C patients [age 48.4 ± 6.7 years, 38.8% female, 16% African American, 60% Obese (BMI > 30), 51% Overweight (BMI > 27.5), 71% genotype 1, 21% cirrhosis, 12% DM, 76% with high pretreatment viral load, and 44% treatment naïve] treated with PEG-IFN+RBV. In this cohort, after a full course of treatment, 76% achieved EVR, 57% achieved cEVR, and 41% achieved SVR. Rates of SVR in genotype 1 were 35% and 55% in non-genotype 1 patients.

Pretreatment depression was seen in 22.4% of the patients. Within this group the prevalence for “Any Depression” (AD) (including those with pre-existing depression and those with new depression during treatment), was 55.22% (*N* = 67). The prevalence for “Treatment-related Depression” (TRD) was 36.54% (*N* = 52). The history of depression was evenly distributed across the treated cohort, regardless of their genotype, gender, pattern of response, as well as presence of cirrhosis, or obesity (Tables 1 and 2) and were not statistically correlated with any of these co-morbidities or demographics.

**Table 1:** Differentially expressed genes in cohorts with “Any Depression” and “New Depression”, where down-regulation is indicated by the color red and up-regulation is indicated by the color blue. Gene abbreviations are as follows: PDGFA (platelet-derived growth factor alpha polypeptide), STAT4 (signal transducer and activator of transcription 4), TGFB1 (transforming growth factor β1), PF4 (platelet factor 4), EP300 (E1A binding protein p300), PRKRIR (repressor of interferon-inducible double-stranded RNA-dependent inhibitor protein-kinase), TRAF6 (TNF receptor-associated factor 6)

Depression model	Prevalence (%)	Differentially expressed genes							
Any Depression	55.22	PDGFA	STAT4	TGFB1	PF4		
		*P* = 0.0054	*P* = 0.0396	*P* = 0.0152	*P* = 0.0123		
New Depression	36.54	PDGFA	STAT4	TGFB1	EP300	PRKRIR	TRAF6
		*P* = 0.0318	*P* = 0.0082	*P* = 0.0194	*P* = 0.0275	*P* = 0.0439	*P* = 0.0142

**Table 2:** Distribution of the prevalence of “Any Depression” across group cohorts

Cohorts	Number of subjects in cohort (% of total enrolled)	Incidence of depression in cohort (% of cohort)
Genotype I	47 (70.1)	28 (59.6)
Female	26 (38.8)	16 (61.5)
Re-treated	37 (55.2)	24 (64.9)
SVR	28 (41.8)	14 (50.0)
Cirrhosis	13 (20.0)	9 (69.2)
Obesity	41 (61.2)	25 (61.0)

SVR, sustained virological response.

### Gene expression data

The mRNA expression profile associated with Any Depression (AD) included four genes, three of them (PDGFA, PF4, and TGF-β1) were down-regulated (*P*-values: <0.0054, <0.0123 and <0.0152; respectively), while the STAT4 gene was up-regulated (*P*-value <0.0396) (Table 2). Gene expression profile associated with TRD included six genes three of them, PDGFA, EP300, and TGF-β1 were down-regulated (*P*-values: <0.0318, <0.0275 and <0.0194; respectively), while PRKRIR, TRAF6, and STAT4, genes were up-regulated (*P*-value <0.0439, <0.0142, <0.0082; respectively) (Table 3).

**Table 3:** Distribution of the prevalence of “Treatment-related Depression” across group cohorts, where the TRD cohort excludes those (*N* = 15) having depression at the start of the study

Cohorts	Number of subjects in cohort (% of total enrolled)	Incidence of depression in cohort (% of cohort)
Genotype 1	37 (71.2)	16 (43.2)
Female	16 (30.8)	4 (25.0)
Re-treated	28 (53.8)	12 (42.9)
SVR	26 (50.0)	10 (38.5)
Cirrhosis	9 (17.6)	5 (55.6)
Obesity	33 (63.5)	16 (48.5)

SVR, sustained virological response.

Using the gene expression profiles, predictive models for both classes of depression were calculated. The best model predicting treatment-related depression included expression levels of TRAF6 and TGF-β1 with a *P*-value of 0.001185, a sensitivity of 63.16% (38.4–83.7%), a specificity of 87.88% (71.8–96.6%), and an area under the curve (AUC) of 0.748 (0.608–0.858).

The predictive model for any depression relied solely on expression levels of TGF-β1 with a *P*-value of 0.01242, a sensitivity of 67.57% (50.2–82.0%), a specificity of 63.33% (43.9–80.1%), and an AUC of 0.642 (0.516–0.756).

## Discussion

Patients with chronic hepatitis C undergoing PEG-IFN+RBV therapy are at an increased risk for developing depression or aggravating pre-existing depression. Several mechanisms for the development of IFN-related depression have been suggested, however, no solid evidence for a common molecular mechanism has yet been proffered. At the same time, markers capable of predicting depression in CH-C patients are highly desirable as active depression during HCV treatment may jeopardize desired therapeutic outcomes and patients' health-related quality of life (Dan et al. 2006; Younossi et al. 2007).

Previous studies in MDD, over the past decade, have increasingly shown the profound involvement of the deregulation of the immune system, including the cytokine network (Maes et al. 1990; Myint and Kim 2003; Schiepers et al. 2005). In particular, events causing activation of the immune system, with the resultant increase in pro-inflammatory cytokines, often coincides with the onset of depression (Maes 1995; Connor and Leonard 1998; Yirmiya 2000; Capuron and Dantzer 2003). In turn, the shift of the T-helper Th1/Th2 balance toward a Th1-type inflammatory response (Maes 1995) occurs in a large number of MDD cases (Myint and Kim 2003; Myint et al. 2005).

Of note, in our study, we observed a significant baseline up-regulation of STAT4 in HCV patients with both a history of depression and new treatment-related depression. This gene has been shown to be intimately involved in the signaling cascade necessary for the activation and consequent pro-inflammatory signal cascade of Th1 type cells (Saraiva et al. 2009).

A recent study on the effects of Th1/Th2 class cytokines on gene expression in cell culture found that Th2 class cytokines up-regulate the prepropeptide PDGF A chain (Lisak et al. 2007). Indeed, in our study, both the presence of “Any Depression” as well as “Treatment-related Depression” resulted in significantly lower expression of PDGFA. These studies point toward a decrease in the Th2 class cytokine signaling as the potential mechanism of depression for these patients. In addition, for HCV patients with “Any Depression”, the PF4 gene was significantly down-regulated. The PF4 encodes for the soluble protein CXCL4, which is directly involved in the up-regulation of Th2 class of cytokines (Romagnani et al. 2005; Mueller et al. 2008). Other genes of interest associated with TRD include TRAF6 and PRKRIR, both of which are involved in inflammatory diseases and in the inflammatory signaling cascade (Cejas et al. 2010; Kuenzel et al. 2010), and PDGFA which encodes the prepropeptide PDGF A chain. PDGFA is specifically up-regulated by Th2 class cytokines (Lisak et al. 2007). In our study, this gene was markedly suppressed, pointing to a decrease in the Th2 class cytokine signaling in HCV patients who develop depression. In fact, our data necessitate a closer examination of the pretreatment baseline levels of Th1 class and Th2 class cytokines in patients scheduled for IFN-α therapy, as interferon-induced depression may in fact involve a pre-existing imbalance in the host Th1/Th2 levels, rendering certain patients vulnerable to depression.

**Figure 1:**
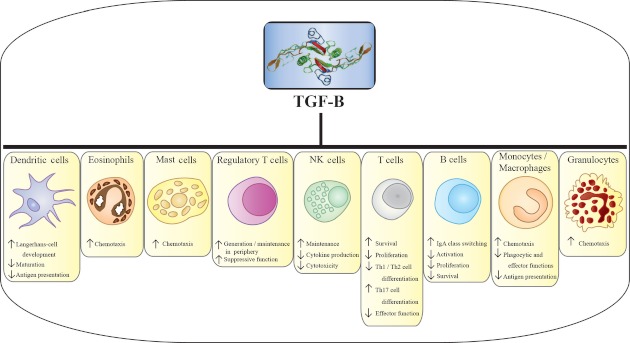
Transforming growth factor-b (TGF-β) and its effects on a large group of secreted cytokines, with a wide range of functional properties.

Our study also supports the potential role of TGF-β1 in IFN-related depression. TGF-β1 is mainly secreted by regulatory T cells such as type 1 regulatory T cells and T-helper type 3 cells (Th3) and is thought to be essential for the maintenance of immune homeostasis and for the suppression of autoimmunity (Groux et al. 1997; Taylor et al. 2006; Zhang et al. 2006). TGF-β1 is known to not only promote T-helper type 2 cell (Th2) differentiation (Barral-Netto et al. 1992) but also to exert a strong inhibitory effect on the production of pro-inflammatory cytokines such as interferons (IFNs), tumor necrosis factor (TNF-α), and IL-2 (Schmitt et al. 1994; Prud'homme and Piccirillo 2000) (Fig. 1). Recent studies indicate that, TGF-β1 plays a role in the development of depression by shifting the balance between the pro-inflammatory/anti-inflammatory cytokines seen in this disorder (Myint et al. 2005; Lee and Kim 2006). In fact, recent studies on MDD have shown that significantly lowered pretreatment TGF-β1 levels in the depressed patients increase following antidepressant therapy (Myint et al. 2005). The decreased baseline levels of TGF-β1 seen within our cohort of HCV patients who ultimately developed depression during treatment, may well follow the same etiology as seen in patients with MDD. Importantly, TGF-β1 has been extensively studied within the context of liver disease, particularly in relation to inflammation and fibrosis (Wynn and Barron 2010). However, little is known about its role within the context of PEG-IFN+RBV treatment of HCV and its associated side effects. The current study is the first to point to TGF-β1 as having a pivotal role in IFN-related depression.

Importantly, worldwide efforts in genome-wide profiling of the polymorphisms associated with MDD and antidepressant treatment outcomes produced only a handful of the candidate genes. Moreover, even in the largest of these studies, the genome-wide significance was not achieved (see Laje and McMahon 2011; Major Depressive Disorder Working Group of the Psychiatric GWAS Consortium 2012, for recent reviews). This observation most probably means that the genetic research in MDD shall proceed by studying smaller, but clearly defined groups of the patients rather than by sheer increase in overall power of the study. Thus, we believe that our approach to the dissection of IFN-α-induced depression may be worthwhile to replicate for other homogenous groups of MDD patients.

In conclusion, our data demonstrate a significant down-regulation of TGF-β1 and dysregulation of Th1-Th2 cytokine balance in the depression associated with IFN-based treatment of HCV infection. We propose that TGF-β1 may play a role in the imbalance of the Th1/Th2 cytokine ratio in patients with CH-C and depression. With further validation, TGF-β1 and other components of Th1/Th2 regulation pathway may provide a quantitative marker for HCV patients predisposed to treatment-related depression.
